# Apolipoprotein C3 is negatively associated with estrogen and mediates the protective effect of estrogen on hypertriglyceridemia in obese adults

**DOI:** 10.1186/s12944-023-01797-0

**Published:** 2023-02-28

**Authors:** Jinman Li, Honglin Sun, Ying Wang, Jia Liu, Guang Wang

**Affiliations:** 1 Department of Endocrinology, Beijing Chao-Yang Hospital, Capital Medical University, No. 8, Gongti South Road, Chaoyang District, Beijing, 100020 China; 2 Department of Medical Examination, Beijing Chao-Yang Hospital, Capital Medical University, Beijing, 100020 China

**Keywords:** APOC3 protein, Estradiol, Estrogen, Triglyceride, Hypertriglyceridemia

## Abstract

**Background:**

Both estrogen and apolipoprotein C3 (ApoC3) play crucial roles in lipid metabolism. But the link between them remains unclear, and it is unknown whether estrogen regulates triglyceride (TG) levels via ApoC3. Researchers hypothesized that estrogen exerts a regulatory effect on ApoC3 metabolism, and that this regulation could play a significant role in lipid metabolism. To explore this potential link, the present investigation aimed to examine the associations between estradiol (E2), ApoC3, and TG levels in both males and females.

**Methods:**

A total of 519 obese people (133 males and 386 premenopausal females) were recruited. Based on their TG levels, the participants were split into two groups [hypertriglyceridemia (HTG) group: TG ≥ 1.7 mmol/L; control group: TG < 1.7 mmol/L]. Serum ApoC3, E2, and TG levels were measured and compared in those two groups for both sexes separately. To ascertain the connection among E2, ApoC3, and TG, linear regression and mediation analysis were used.

**Results:**

Participants in the HTG group presented higher levels of ApoC3 (*P* < 0.001). In contrast, they tend to have lower E2 levels than the control. Linear regression analysis proposed that in both sexes, E2 was negatively associated with ApoC3 levels. The relationship remained significant after adjustment for confounding factors (male: standardized *β* = -0.144, t = -2.392, *P* < 0.05; female: standardized *β* = -0.077, t = -2.360, *P* < 0.001). Furthermore, mediation analysis revealed the relationship between reduced E2 levels and elevated TG levels is directly mediated by ApoC3.

**Conclusions:**

In obese men and premenopausal women, ApoC3 was negatively and linearly correlated with serum E2 levels. The findings showed that estrogen may suppress ApoC3 expression and thus lower TG levels.

**Supplementary Information:**

The online version contains supplementary material available at 10.1186/s12944-023-01797-0.

## Background

Numerous clinical studies have shown that premenopausal women are less likely to develop coronary artery disease, diabetes, obesity, and dyslipidaemia than men [1]. In comparison to men, women have acute myocardial infarction about a decade later [2]. Furthermore, at the same age, women are 50% less likely than males to have acute cardiovascular disease (CVD) [2, 3]. Endogenous estrogen is thought to be a mechanism for differentiating this risk. It can regulate lipid metabolism and control serum lipoprotein levels [4]. Elevated endogenous estrogen levels are significantly associated with reduced low-density lipoprotein (LDL) and low triglyceride (TG) levels, which are strongly related to CVD and metabolic syndrome [3, 5]. This effect is particularly prominent in obese people, who have higher risks of developing diseases [6–8].

Estradiol (E2) is a steroidal estrogen [9]. The biosynthesis of E2 is a multi-step process that involves the conversion of cholesterol into pregnenolone, a 19-carbon steroid hormone [10]. Pregnenolone is then metabolized into testosterone, which is further converted into the primary estrogens, estrone and 17β-estradiol [10]. E2 is the most potent form of mammalian estrogenic steroids [9].

Apolipoprotein C3 (ApoC3) has recently been a hot topic of research. It is increasingly considered a vital metabolic regulator of human triglyceride-rich lipoprotein (TRL) [11]. ApoC3 is mainly synthesized in the liver [12]. It not only inhibits the hydrolysis of TRL by controlling lipoprotein lipase but also suppresses the uptake of TRL residues by the liver [13]. Furthermore, high concentrations of ApoC3 affect the activity of hepatic lipase [14], which leads to impaired conversion of very-low-density lipoproteins (VLDL) to intermediate-density lipoproteins (IDL) and LDL [15]. All of these factors can contribute to the accumulation of atherogenic VLDL and chylomicron residues [16]. A growing number of trials have demonstrated that lower ApoC3 levels could reduce the risk of CVD [17]. In comparison to younger women, older women, particularly postmenopausal women, showed greater levels of ApoC3 [18]. Additionally, researchers noticed that men had higher levels of ApoC3 than women [18, 19].

Since both estrogen and ApoC3 play critical roles in lipid metabolism, the paper hypothesises that estrogen affects ApoC3 metabolism. However, few studies have focused on the correlation between estrogen and ApoC3 levels in men and premenopausal women, which would have enormous implications for the general population.

The current study evaluated the potential sex-specific relationship between E2 and metabolic parameters in obese people.

## Method

### Research population

This cross-sectional study included obese patients (BMI ≧ 30.0 kg/m2) who underwent routine medical checkups at the Beijing Chaoyang Hospital from June 2017 to March 2021. All included women were premenopausal. Individuals with major chronic illnesses such as severe CVD, liver or renal function impairment, systemic inflammatory disease, infectious disease, or cancer were excluded. The exclusion criteria also included using any medication that affects estrogen, glucose, or lipids; missing detailed data; or outliers. Ultimately, 519 participants were recruited. The Ethics Committee of Beijing Chaoyang Hospital, Capital Medical University, approved the study protocol. Written informed consent was received by all subjects before the study.

### Anthropometric and biochemical measurements

To gather information on the patients' health and medications, researchers employed a standard questionnaire. Height, weight, waist circumference (WC), systolic blood pressure (SBP), and diastolic blood pressure (DBP) were measured at baseline. A stationary stadiometer with a movable headboard was used to measure height to the closest 0.1 cm. Weight was accurately measured to the closest 0.1 kg on the weighing scale while participants were clothed (without shoes) and indoors. WC was surveyed at the narrowest part of the torso to the nearest 0.1 cm by trained staff using tape measures. Blood pressure was measured twice after 10 min of lying down, and the average of the two results was taken as the patient's blood pressure level. The formula for calculating BMI was BMI = [weight (kg)/height^2^ (m^2^)].

Samples of venous blood were taken after an overnight fast. And at -80 °C, the samples were stored. Since previous study has suggested that there is no significant difference in TG and apolipoprotein B levels between the luteal and follicular phases of non-menopausal women, based on statistical analysis of the data [20]. All females had their blood drawn outside of their menstrual period. Total cholesterol (TC), TG, high-density lipoprotein cholesterol (HDL-C), low-density lipoprotein cholesterol (LDL-C), ApoC3, and apolipoprotein C2 (ApoC2) levels were assessed by colorimetric enzymatic assays using an autoanalyzer (Hitachi 7170). hypertriglyceridemia (HTG) is defined as TG ≧ 1.7 mmol/L as recommended [21]. Dyslipidemia was defined as HDL-C < 1.0 mmol/L, LDL-C ≧ 3.37 mmol/L, TC ≧ 5.2 mmol/L, TG ≧ 1.7 mmol/L, or a self-reported previous diagnosis of hyperlipidemia. E2, total testosterone (TT), and progesterone (P) were measured by chemiluminescent immunoassay using the Kikuchi 1000 immunoassay (Siemens). Fasting blood glucose (FBG), fasting insulin (FINS), and C-peptide levels were tested at Beijing Chao-yang Hospital’s central chemistry laboratory. The glucose oxidase method was used to measure plasma FBG, whereas the chemiluminescence method was used to measure FINS.

### Statistical analysis

The statistics software IBM SPSS, version 26, was used to conduct the study's statistical analysis. To explore the gender specificity of ApoC3 levels, researchers performed separate analyses for male and female participants. To examine the normality of the variables, the Shapiro–Wilk test was used. The skewed distribution of the data required log-transformation for TG, ApoC3, E2, and TT. The t test was used to analyse continuous parameters having a normal distribution. The results are displayed as the mean ± standard deviation. nonparametric tests were used to analyse continuous parameters with nonnormal distributions. The outcomes are expressed as medians and upper and lower quartiles. Data for categorical variables are expressed as numbers (%). Gender-specific Spearman and Pearson correlation analyses were performed to assess the relationship between ApoC3 (dependent variable) and sex hormones (independent variable). Linear regression analysis was used to assess the correlations, and 95% confidence intervals (CI) were used for statistical inference. The significant statistical threshold was established at 0.05. Standardized coefficients *β* and t values were used to describe the results. Variables with no covariance were selected for adjustment. Model 1 was unadjusted, model 2 had age and BMI adjustments, whereas model 3 had adjustments for age, ApoC2, BMI, FBG, and C-peptide. Finally, mediation analysis was utilized to investigate the part that ApoC3 played in the association between E2 and TG after controlling for age, ApoC2, BMI, C-peptide, and FBG levels. First, TG was considered the outcome, and E2 had a coefficient, c, as the total effect on TG (TG = c × E2 + control variables + e1). Then, ApoC3 was added to the model as a mediator (TG = c′ × E2 + b × mediator + control variable + e2). Finally, regression analysis with ApoC3 and E2 (mediators = a × E2 + control variable + e3) was performed. The mediation impact was not recorded if ‘c’, ‘a’, or ‘b’ was insignificant. If ‘c’’ is nonsignificant, a fully mediated effect is considered [22, 23]. All models were revalidated by bootstrap testing.

## Results

### Clinical characteristics of study participants in males and females

The baseline characteristics of the 519 participants (133 males and 386 females) with HTG and without HTG are presented in Table 1.

**Table 1:** Baseline characteristics of males and females with and without hypertriglyceridemia (HTG)

Variables		Non-HTG (*n* = 191)	HTG (*n* = 228)	*P* value
**Male**	**N**	63	70	–
	**Age, y**	31.65 ± 9.63	31.94 ± 7.77	0.847
	**SP, mmHg**	132 ± 16.08	126.26 ± 10.6	0.215
	**BP, mmHg**	81.38 ± 7.46	83.16 ± 7.2	0.478
	**BMI, kg/m2**	**44.85 ± 8.84**	**41.92 ± 7.13**	**0.036**
	**WC, cm**	129.91 ± 15.02	126 ± 15.34	0.164
	**FBG, mmol/L**	**5.98 ± 1.33**	**7.4 ± 3.82**	**0.004**
	**FINS, uIU/mL**	34.98 ± 21.35	38.26 ± 23.44	0.417
	**C-Peptide, ng/mL**	**4.63 ± 1.49**	**5.23 ± 1.83**	**0.048**
	**TC, mmol/L**	**4.63 ± 0.8**	**5.43 ± 1.02**	< **0.001**
	**TG, mmol/L**	**1.24 ± 0.26**	**3.93 ± 4.16**	< **0.001**
	**HDL-C, mmol/L**	0.97 ± 0.15	0.98 ± 0.18	0.709
	**LDL-C, mmol/L**	3.14 ± 0.65	3.37 ± 0.75	0.06
	**ApoC3, mg/dL**	**7.03 ± 1.58**	**15.77 ± 8.75**	< **0.001**
	**ApoC2, mg/dL**	**2.75 ± 0.92**	**5.19 ± 1.48**	< **0.001**
	**TT, nmol/L**	8.97 ± 4.17	8.9 ± 2.97	0.563
	**E2, pmol/L**	198.94 ± 86.53	180.18 ± 63.92	0.249
	**PRG, ng/mL**	0.60 (0.44, 0.89)	0.61 (0.46, 0.78)	0.924
	**OC, ug/L**	20.01 ± 6.68	22.27 ± 7.73	0.185
**Female**	**N**	128	158	–
	**Age, y**	32.25 ± 8.23	33.04 ± 7.74	0.346
	**SBP, mmHg**	127.57 ± 13.88	130.2 ± 16.21	0.309
	**DBP, mmHg**	82.57 ± 10.25	84.91 ± 11.5	0.212
	**BMI, kg/m2**	38.08 ± 6.25	38 ± 6.2	0.907
	**WC, cm**	111.17 ± 14.65	112.56 ± 13.56	0.371
	**FBG, mmol/L**	**5.68 ± 1.31**	**6.83 ± 2.79**	< **0.001**
	**FINS, uIU/mL**	27.16 ± 16.35	30.41 ± 16.22	0.061
	**C-Peptide, ng/mL**	**3.84 ± 1.23**	**4.38 ± 1.43**	< **0.001**
	**TC, mmol/L**	**4.6 ± 0.74**	**5.12 ± 0.87**	< **0.001**
	**TG, mmol/L**	**1.23 ± 0.3**	**2.58 ± 1.29**	< **0.001**
	**HDL-C, mmol/L**	**1.16 ± 0.29**	**1.04 ± 0.19**	< **0.001**
	**LDL-C, mmol/L**	**2.95 ± 0.56**	**3.29 ± 0.67**	< **0.001**
	**ApoC3, mg/dL**	**8.09 ± 2.05**	**13.05 ± 4.37**	< **0.001**
	**ApoC2, mg/dL**	**3.04 ± 0.94**	**4.94 ± 1.75**	< **0.001**
	**TT, nmol/L**	2.1 ± 0.95	1.99 ± 0.97	0.157
	**E2, pmol/L**	**345.4 ± 263.14**	**303.82 ± 294.21**	**0.047**
	**PRG, ng/mL**	0.79 (0.57, 9.39)	0.71 (0.47, 1.40)	0.171
	**OC, ug/L**	21.08 ± 7.39	20.45 ± 8.68	0.299

Data are presented as the mean ± SD or median (upper and lower quartiles) or number. ApoC2, ApoC3, E2, TG, and TES were log-transformed due to a skewed distribution. *ApoC2* apolipoprotein C2, *ApoC3* apolipoprotein C3, *BMI* body mass index, *DBP* diastolic blood pressure, *E2* estradiol, *FINS* fasting insulin, *FBG* fasting blood glucose, *HDL-C* high-density lipoprotein cholesterol, *LDL-C* low-density lipoprotein cholesterol, *OC* osteocalcin, *SBP* systolic blood pressure, *TC* total cholesterol, *TT* testosterone, *TG* triglycerides, *PRG* progesterone, *WC* waist circumference

In comparison to controls, the HTG group had higher FBG and C-peptide levels among both sexes (all *P* < 0.05). In terms of indicators associated with lipid metabolism, the HTG group had higher levels of TC, ApoC2, and ApoC3 (all *P* < 0.001) across both sexes. In females, LDL-C levels were greater in the HTG group whereas HDL-C levels were lower (all *P* < 0.001). Females with HTG showed lower E2 levels than the control group (*P* < 0.05). Men with HTG tended to have lower levels of E2 and LDL-C, even though the difference was not statistically significant (E2: *P* = 0.249; LDL-C: *P* = 0.06). Males and females in the two groups did not greatly vary in terms of age, blood pressure, waist size, or osteocalcin (OC) values. According to earlier studies, estrogen consistently adversely affects TG levels [3], whereas ApoC3 positively regulates TG levels [11]. It is certainly worthwhile to study the relationship between estrogen and ApoC3 levels.

### The correlations between ApoC3 and clinical parameters in all participants

To investigate the link between circulating ApoC3 levels and clinical parameters, researchers categorized the sample by sex and carried out separate bivariate correlation analyses (Table 2).

**Table 2:** The correlation between ApoC3 and clinical parameters in all participants

	**ApoC3** (Male)	**ApoC3** (Female)
Age	0.11	0.079
BMI	-0.29**	-0.102*
WC	-0.196	-0.055
FBG	0.311**	0.270**
C-peptide	0.135	0.089
TC	0.504**	0.474**
TG	0.942**	0.846**
HDL-C	0.066	0.032
LDL-C	0.207*	0.334**
ApoC2	0.767**	0.795**
E2	-0.206*	-0.175**
TT	0.038	-0.064
PRG	0.015	-0.205*

*ApoC2* apolipoprotein C2, *ApoC3* apolipoprotein C3, *BMI* body mass index, *E2* estradiol, *FINS* fasting insulin, *FBG* fasting blood glucose, *HDL-C* high-density lipoprotein cholesterol, *LDL-C* low-density lipoprotein cholesterol, *TC* total cholesterol, *TT* testosterone, *TG* triglycerides, *PRG* progesterone, *WC* waist circumference

ApoC2, ApoC3, E2, TG, and TT were log-transformed due to a skewed distribution. The relationship between PRG and ApoC3 was established by Spearman correlation analysis. Pearson correlation analysis was used to describe the association between ApoC3 and other markers

^*^P < 0.05

^**^P < 0.01

**Fig. 1:**
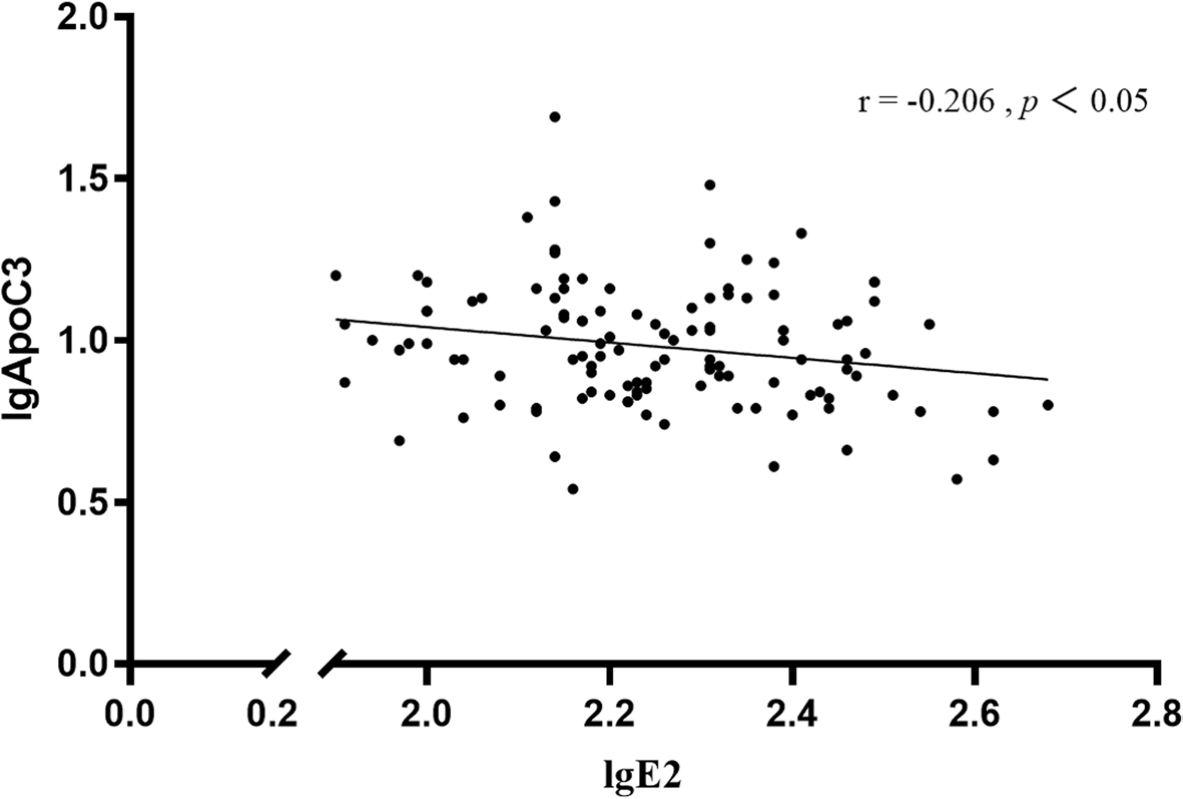
The correlation between ApoC3 levels (dependent variable) and E2 levels (independent variables) among males with obesity. Legends: ApoC3 and E2 were log-transformed due to a skewed distribution. ApoC3 was negatively correlated with the levels of E2 in males (*r* = -0.206, *P* < 0.05)

**Fig. 2:**
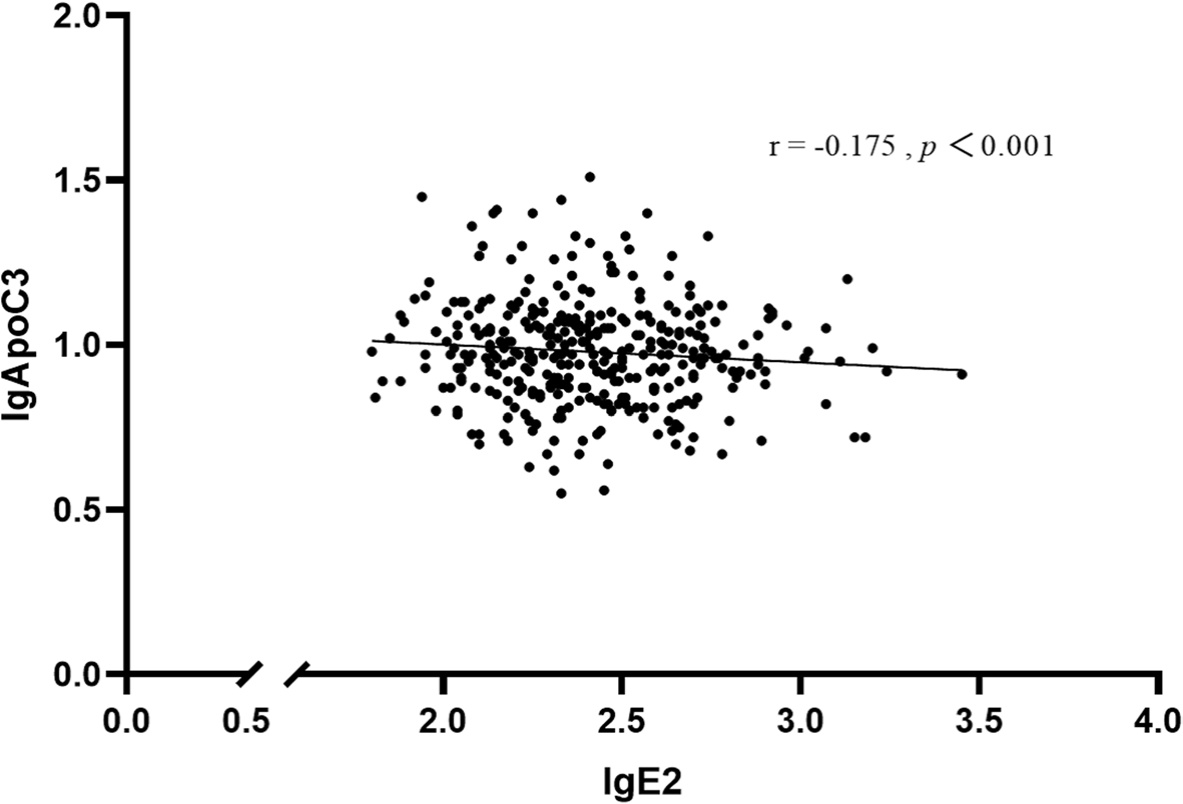
The correlation between ApoC3 levels (dependent variable) and E2 levels (independent variables) among premenopausal females with obesity. Legends: ApoC3 and E2 were log-transformed due to a skewed distribution. ApoC3 was negatively correlated with the levels of E2 in premenopausal females (*r* = -0.175, *P* < 0.001)

ApoC3 was positively correlated with the levels of ApoC2, FBG, LDL-C, TC, and TG. Similar correlations were observed in the gender-separated analysis (all *P* < 0.01). ApoC3 levels were shown to be adversely linked with E2 (male: *r* = -0.206, female: *r* = -0.175, all *P* < 0.05) and PRG (male: *r* = -0.015, *P* = 0.919, female:* r* = -0.205, *P* < 0.05). According to the findings, HTG and ApoC3 levels are positively correlated. In addition, ApoC3 levels were inversely correlated with E2 levels in both males (Fig. 1) and females (Fig. 2).

The relationship between ApoC2 and clinical indicators was evaluated, too. The results are displayed in the supplementary chart (Supplementary Table 1). E2 is not related to ApoC2 levels.

### Association of ApoC3 with serum E2 levels by linear regression analysis

To further explore the relationship between ApoC3 and E2 levels, a linear regression analysis was employed (Table 3).

**Table 3:** Linear regression analysis for the association between ApoC3 levels (dependent variable) and E2 levels (independent variables) among individuals with obesity

	**Standardized *β***	**t**	***P* value**	**95%CI**
**Male**				
Model 1	-0.206	-2.307	0.023	-0.510, -0.039
Model 2	-0.137	-1.510	0.134	-0.422, + 0.057
Model 3	-0.144	-2.392	0.018	-0.353, -0.033
**Female**				
Model 1	-0.175	-3.371	0.001	-0.151, -0.040
Model 2	-0.180	-3.455	0.001	-0.154, -0.042
Model 3	-0.077	-2.360	0.019	-0.077, -0.007

ApoC2, ApoC3, and E2 were log-transformed due to a skewed distribution

Model 1: Crude model

Model 2: adjusted for age and BMI

Model 3: adjusted for age, BMI, FBG, C-peptide, and ApoC2

Serum E2 levels were shown to be inversely associated with ApoC3 levels. This negative association remained after correcting for all nonlinear confounding variables. This relationship was observed in both sexes. However, the association was stronger among males (male: standardized *β* = -0.144, t = -2.392, *P* < 0.05; female: standardized *β* = -0.077, t = -2.360, *P* < 0.001).

### Association of ApoC3 with serum E2 levels by linear regression analysis in participants with or without dyslipidemia

It is fruitful to look into the relationship between ApoC3 and E2 among participants with dyslipidemia because estrogen and blood lipid metabolism are closely related. Participants with or without dyslipidemia were subjected to subgroup analysis by researchers. In both males and females with dyslipidemia, E2 was shown to be inversely correlated with ApoC3 in Supplementary Table 2 (male: standardized *β* = -0.195, t = -2.036, *P* < 0.05; female: standardized *β* = -0.150, t = -2.364, *P* < 0.05). The relationship still existed after correcting for confounding variables (male: standardized *β* = -0.167, t = -2.688, *P* < 0.01; female: standardized *β* = -0.089, t = -2.331, *P* < 0.05). This negative correlation, though, was not significant in the control group for either gender.

### Serial mediation model for a hypothesized pathway to a hypertriglyceridemia event in both sexes

**Fig. 3:**
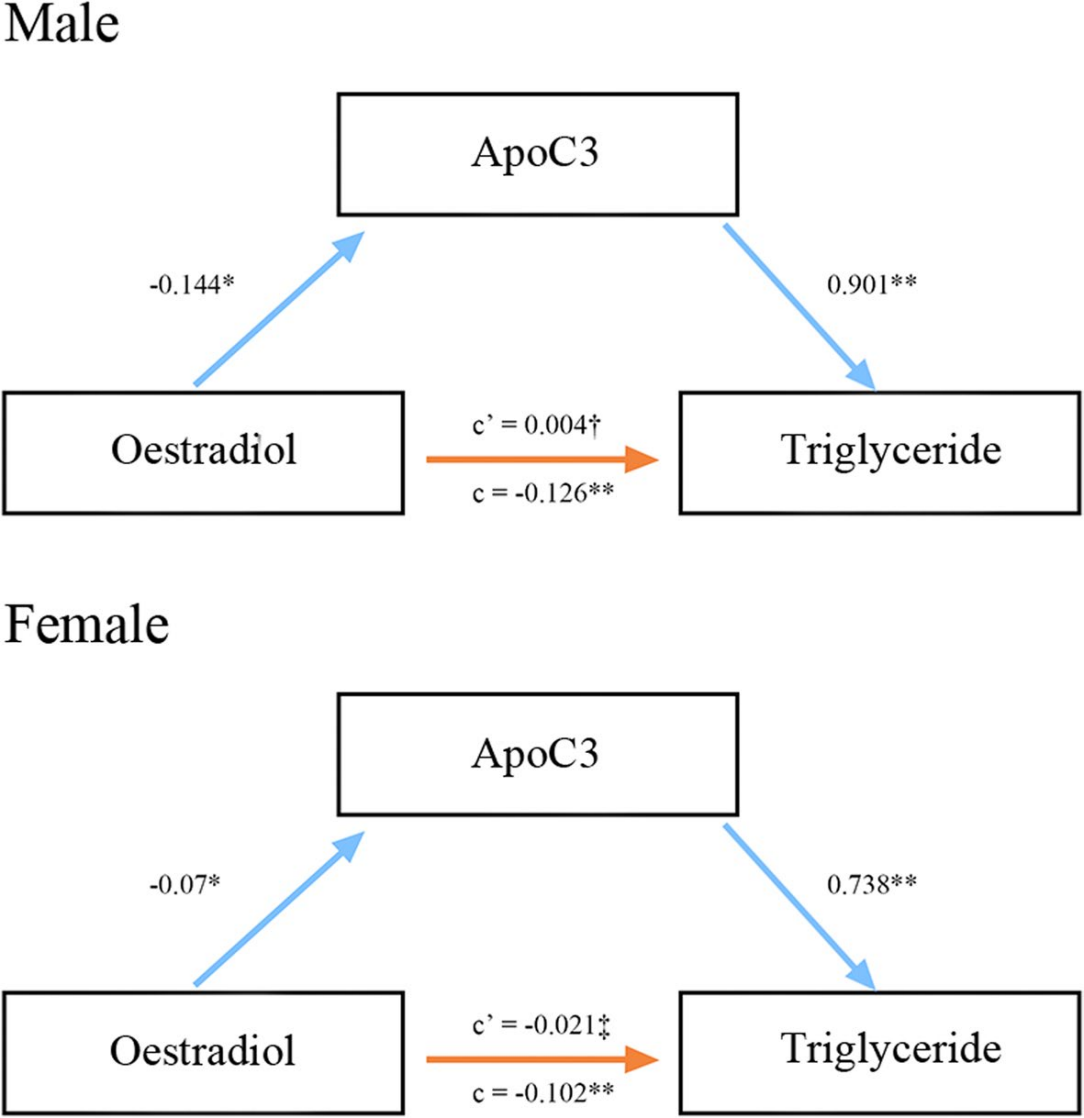
Serial mediation model for a hypothesized pathway to a hypertriglyceridemia event in both sexes. Legends: In this figure, c represents the total effect of the E2 level on hypertriglyceridemia, and c' is the residual effect of the E2 level on hypertriglyceridemia (independent of mediating effects). All analyses incorporated age, BMI, FBG, C-peptide, and ApoC2 as covariates. †*P* = 0.898, ‡*P* = 0.401, *P < 0.05, **P < 0.01

In both men and women, ApoC3 significantly mediated the relationship between E2 and TGs, accounting for 100% of the total effect. Mediation analysis supported the hypothesis that decreased E2 levels led to elevated TG levels by upregulating ApoC3 expression directly (Fig. 3).

The outcomes of the mediation analysis were validated by bootstrapping analysis, and the results were consistent with those of the stepwise method.

## Discussion

This study investigated the association between serum E2 and ApoC3 levels among individuals with obesity from China. It found that ApoC3 was negatively associated with E2 levels in both men and premenopausal women. The mediation analysis indicated that decreased E2 levels led to increased TG levels by increasing ApoC3 levels, by a straight pathway. This finding implied that increased TG levels due to decreasing serum E2 levels may be mediated by ApoC3. This may be one of the pathways through which estrogen affects CVD and other metabolic diseases.

Extensive epidemiological evidence and basic studies have suggested that elevated levels of ApoC3 are strongly associated with CVD [17, 24]. Apoc3 is a secreted glycoprotein generated by the liver that plays a critical role in TRL metabolism [24]. It has been shown to enhance VLDL production from isolated hepatocyte cultures [25]. ApoC3 can cause severe HTG by inhibiting LPL activity [13]. In addition, ApoC3 could inhibit TRL lipoprotein clearance by the liver [26, 27]. Therefore, VLDL and celiac particles would accumulate. It was reported that the loss of function of ApoC3 could confer cardiovascular protection [28–30]. Consistent with this, our study showed that the HTG group had higher ApoC3 levels than the control group, among individuals with obesity. This effect of ApoC3 on lipid metabolism increases the risk of CVD.

Estrogen regulates lipid metabolism in a significant way, and its fluctuations in perimenopausal and postmenopausal women can lead to dyslipidaemia, such as elevated TGs [4]. Studies have reported that TG levels are significantly higher in postmenopausal women than in premenopausal women [31] and that TG levels are significantly lower after treatment with transdermal E2 [32]. There is no clear mechanism to explain how inhibition is mediated. Scholars proposed that estrogen can work directly in the liver to reduce TG content via estrogen receptor (ER) [33]. Animal experiments have evidenced that the ability of hepatic steatosis was lost with the absence of hepatic ER after estrogen reduction [32]. Estrogen reduces de novo fat synthesis in the liver by maintaining acetyl-coa carboxylase (ACC) phosphorylation [34] and promoting free fatty acids (FA) oxidation [35], too. Estrogen also promotes the uptake of FA in adipose tissue [36] and accelerates FA consumption in muscle tissue [37], thus limiting FA transport to the liver and reducing TG production at the source. These effects lead to a decrease in TGs production. In addition, estrogen can regulate serum lipoprotein levels [38]. Therefore, the study speculate that estrogen's inhibitory effect on triglycerides is mediated through the inhibition of ApoC3. Few studies have focused on the correlation between estrogen and ApoC3.

In our study, the HTG group had lower E2 levels in females, as described above. However, this difference was not significant in men. The results could be attributed to interference from confounding factors. After adjusting for confounding factors, a robust association was observed between E2 and TG levels, in both sexes. In order to comprehensively examine the correlation between E2 and ApoC3, we conducted an analysis of this relationship in both normolipidemic and hyperlipidemic patients. In males and females with dyslipidemia, a significant inverse correlation was observed between E2 and ApoC3. Conversely, in the control group, this negative correlation did not reach statistical significance for either gender. The present findings suggest that the regulatory impact of E2 on ApoC3 expression may be selectively induced in the context of dyslipidemia. Nevertheless, additional investigations are warranted to more fully characterize the complex interplay between these factors and the underlying biological pathways involved.

Individuals with obesity (BMI ≥ 30.0 kg/m^2^) have an elevated risk of developing cardiovascular and metabolic disorders [39]. Therefore, to achieve greater precision in our findings, the researchers conducted a cross-sectional analysis specifically among individuals with obesity.

The present investigation demonstrated a consistent linear association between serum E2 levels and ApoC3 in both men and premenopausal women, which persisted even after adjustment for confounding variables. This relationship was further observed to be more prominent among individuals with dyslipidemia. Additionally, the results of the mediation analysis supported the hypothesis that estrogen may mitigate TG levels by suppressing ApoC3 expression.

For the first time, the current study proposed a link between estrogen, ApoC3, and TGs in premenopausal women and men. This offers fresh perspectives on heart disease treatment and prevention in the future. To uncover the underlying mechanisms, additional research is required.

### Comparisons with other studies and what does the current work add to the existing knowledge

Prior research on estrogen concentrated on TC, TG, and LDL-C, which are common clinical markers of lipid metabolism. Few research has been conducted to investigate the link between estrogen and ApoC3. According to our clinical research, estrogen may suppress the expression of ApoC3, which would lower TG levels.

### Study advantages and limitations

This study's innovation is its main strength. We used mediation analysis to show how estrogen, ApoC3, and TGs are related. The study's strengths also include standardised measurement laboratory data and thorough information on drug intake.

Several limitations exist in this research. First, blood was gathered before the women's periods because E2 levels might fluctuate over the menstrual cycle. However, the menstrual cycle was not standardized, which may have resulted in some bias. Furthermore, this present trial is a small sample cross-sectional study involving Chinese individuals. This may restrict the generalizability of current findings to populations in other locations; hence, more research with large samples in multiregional cohorts is needed.

## Conclusion

In summary, ApoC3 was negatively and linearly associated with serum E2 levels in men and premenopausal women with obesity. This implies that estrogen may suppress ApoC3 expression and thus lower TG levels. Our findings provide new insights into the prevention and management of heart disease in the future.

## Supplementary Information

**Additional file 1.** **Supplementary Table 1.** The correlation between ApoC2 and clinical parameters among all participants. 

**Additional file 2. Supplementary Table 2. **Liner regressionanalysis for the association of ApoC3 levels (dependent variable) and E2 levels(independent variables) with or without dyslipidemia. 

## Data Availability

The datasets used to support this study are not freely available due to participants’ privacy protection. The data can be obtained from the corresponding author upon reasonable request.

## Abbreviations

ACC: Acetyl-coa carboxylase
ApoC2: Apolipoprotein C2
ApoC3: Apolipoprotein C3
BMI: Body mass index
CI: Confidence intervals
CVD: Cardiovascular disease
DBP: Diastolic blood pressure
E2: Estradiol
ER: Estrogen receptor
FA: Free fatty acids
FINS: Fasting insulin
FBG: Fasting blood glucose
HDL-C: High-density lipoprotein cholesterol
IDL: Intermediate-density lipoproteins
LDL: Low-density lipoprotein
LDL-C: Low-density lipoprotein cholesterol
OC: Osteocalcin
SBP: Systolic blood pressure
TC: Total cholesterol
TRL: Triglyceride-rich lipoprotein
TT: Testosterone
TG: Triglyceride
PRG: Progesterone
VLDL: Very-low-density lipoproteins
WC: Waist circumference
